# GIP contributes to postprandial regulation of splanchnic blood supply in humans with type 2 diabetes: a randomised, single-blinded, placebo-controlled, crossover study

**DOI:** 10.1007/s00125-026-06788-1

**Published:** 2026-07-02

**Authors:** Rasmus S. Rasmussen, Sophie W. Nielsen, Lotte Alstrup, Tanne S. W. Larsson, Jonathan K. Hansen, Rune P. Pedersen, Mark B. Vestergaard, Bryan Haddock, Helle H. Johannesen, Henrik B. W. Larsson, Jens J. Holst, Bolette Hartmann, Mette M. Rosenkilde, Ali Asmar, Ulrik B. Andersen, Lærke S. Gasbjerg

**Affiliations:** 1 Department of Biomedical Sciences, Faculty of Health and Medical Sciences, University of Copenhagen, København, Denmark; 2 Department of Clinical Physiology and Nuclear Medicine, Rigshospitalet, Glostrup, Denmark; 3 Novo Nordisk Foundation Center for Basic Metabolic Research, Faculty of Health and Medical Sciences, University of Copenhagen, København, Denmark; 4 Department of Clinical Physiology and Nuclear Medicine, Bispebjerg, København, NV Denmark; 5 Department of Clinical Medicine, Faculty of Health and Medical Sciences, University of Copenhagen, Copenhagen, Denmark

**Keywords:** Endocrinology, GIP, Gut hormones, Haemodynamic, Incretin hormones, Postprandial metabolism

## Abstract

**Aims/hypothesis:**

In healthy lean humans, endogenous glucose-dependent insulinotropic polypeptide (GIP) contributes significantly to the postprandial increase in arteria mesenterica superior blood flow. The vascular biology related to activation of the GIP receptor is markedly impaired in individuals with type 2 diabetes and is sometimes absent. In this population, we investigated the role of endogenous GIP on postprandial splanchnic blood flow by using the GIP receptor antagonist, GIP(3-30)NH_2_. The primary outcome of this study was the changes in blood flow in arteria mesenterica superior during oral glucose with or without GIP receptor antagonist infusion.

**Methods:**

Ten participants with type 2 diabetes (age 20–80 years, BMI 20–35 kg/m^2^, and HbA_1c_ >48 mmol/mol and <75 mmol/mol) were investigated in a randomised, placebo-controlled, crossover study. On four separate occasions, participants received the following treatment: oral glucose + i.v. GIP(3-30)NH_2_; oral glucose + i.v. saline (154 mmol/l NaCl); oral water + i.v. GIP(3-30)NH_2_; oral water + i.v. saline. Participants were randomly assigned to intervention groups using (random.org). Participants were unaware of allocation, while investigators were aware. No additional allocation concealment procedures were used. During all four interventions, splanchnic blood flow was measured using phase-contrast MRI in the arteria mesenterica superior, truncus coeliacus and vena portae during oral glucose (75 g) or water ingestion. The study was conducted at Rigshospitalet, Copenhagen. Liver volume and oxygenation, as well as gallbladder volume, were assessed. Blood samples were collected and analysed for insulin, C-peptide, GIP, glucagon and glucose.

**Results:**

Oral glucose alone increased mean blood flow in arteria mesenterica superior by 57% (95% CI 26, 88) and this was 15% (95% CI −2, 32) lower during concomitant GIP receptor antagonist infusion, *p*=0.012. Infusion of GIP receptor antagonist during oral glucose treatment did also result in lower insulin secretion, C-peptide and C-peptide/glucose ratio compared with saline infusion, whereas glucagon levels and plasma glucose were unaffected. Oral water did not affect any outcomes.

**Conclusions/interpretation:**

Endogenous GIP contributes to postprandially increased splanchnic blood flow in people with type 2 diabetes.

**Trial registration:**

ClinicalTrials.gov NCT06426823

**Funding:**

This work was supported by the Novo Nordisk Foundation.

**Graphical Abstract:**

**Figure:**
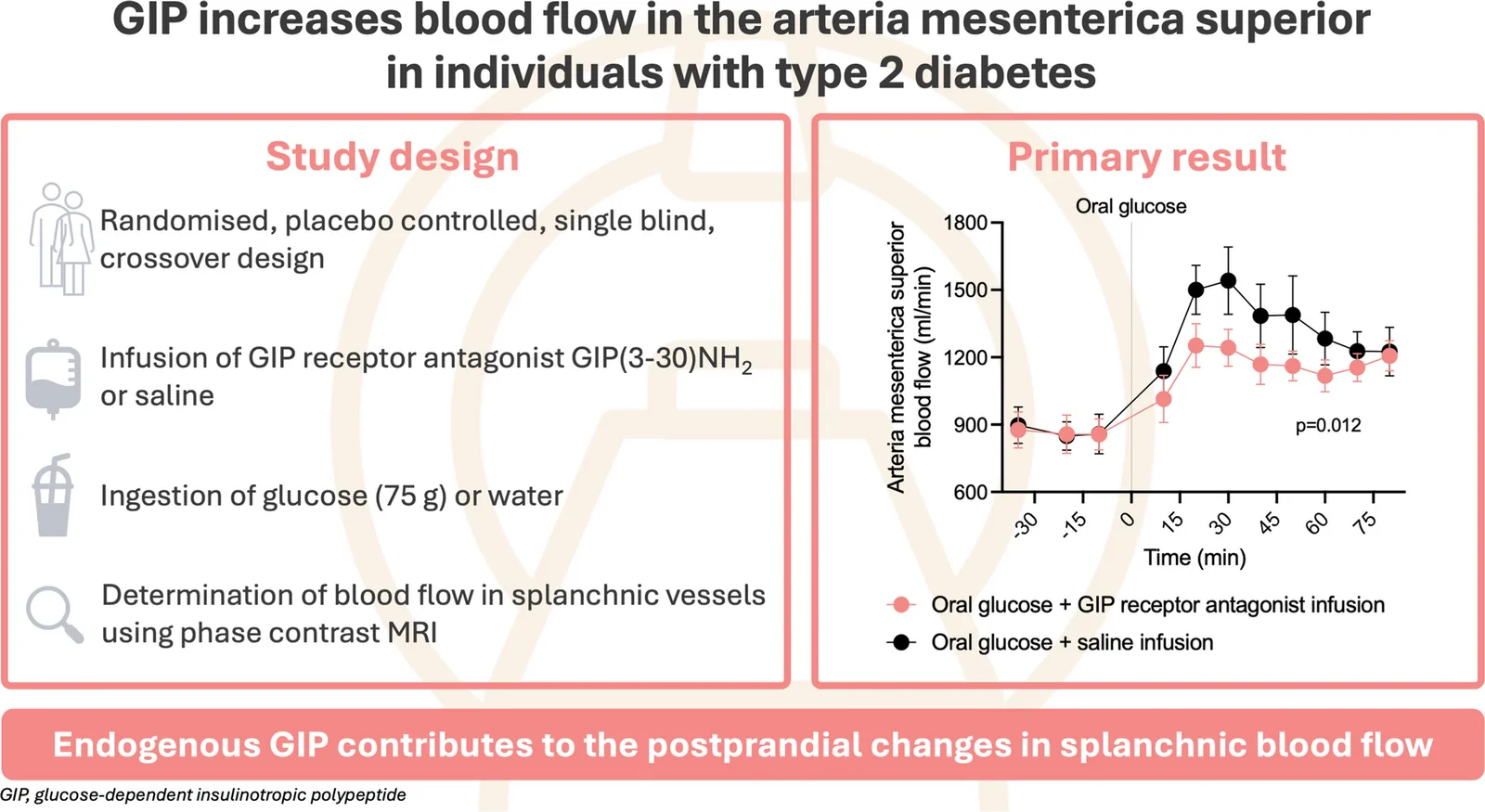

**Supplementary Information:**

The online version contains peer-reviewed but unedited supplementary material available at https://doi.org/10.1007/s00125-026-06788-1.

**Figure:**
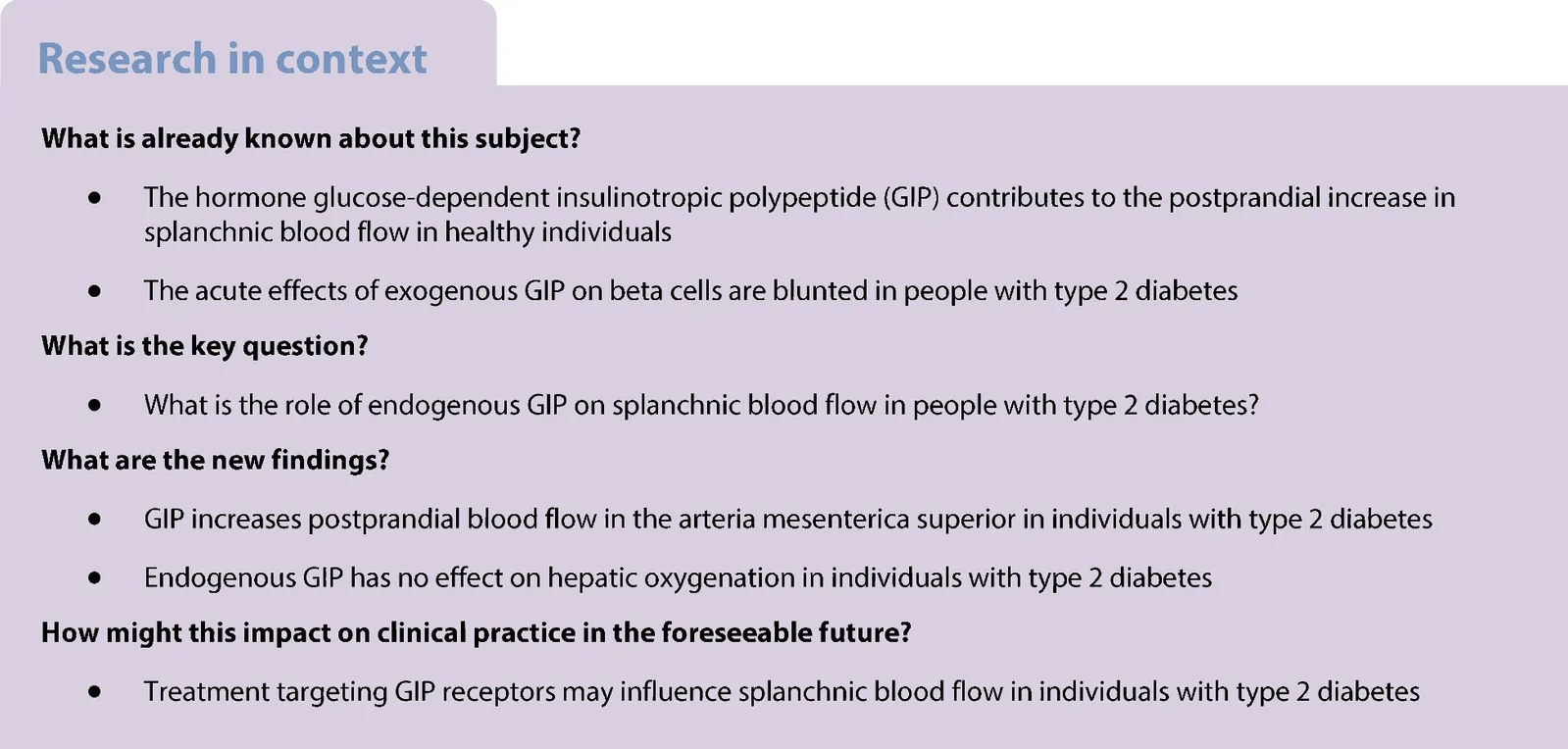

## Introduction

After ingestion of a meal, blood flow increases to the splanchnic area to facilitate nutrient uptake [1, 2]. In healthy individuals, oral glucose ingestion elicits a marked splanchnic haemodynamic response, characterised by a ∼70% increase in arteria mesenterica superior blood flow and a ∼64% increase in vena portae blood flow relative to fasting levels [2, 3]. The increase is thought to be induced by several factors: the direct effect of nutrient and fluid absorption; activity of the enteric nervous system; local non-metabolites that act as vasoactive mediators; and local metabolic vasoactive mediators [4, 5]. In addition, the release of gastrointestinal hormones has been proposed to contribute, including glucose-dependent insulinotropic polypeptide (GIP) secreted by the enteroendocrine K cells [6]. Together with glucagon-like peptide 1 (GLP-1) secreted by the enteroendocrine L cells, GIP is responsible for the incretin effect [7], which potentiates glucose-induced insulin secretion through G_αs_ protein-coupled receptor signalling [8]. Among other functions, GIP infusion, in combination with insulin, markedly increases (fivefold) adipose tissue blood flow (ATBF) and enhances triacylglycerol storage [9, 10] via local capillary recruitment [11]. In overweight and insulin-resistant individuals, GIP fails to increase ATBF [12]. In addition, the insulinotropic effects of GIP and its vascular biology in people with type 2 diabetes is markedly impaired [13, 14]. In individuals with type 2 diabetes and inadequate glycaemic management, the ability of endogenous GIP to enhance insulin secretion is attenuated [15]. Thus, impaired GIP-induced potentiation of insulin secretion may contribute to a compounded reduction in haemodynamic effects through given insulin’s vasodilatory properties [16]. Yet, improved glycaemic regulation appears to partially restore the insulinotropic effect of GIP [17]. Treatment with a long-acting GIP receptor agonist, however, has been shown to augment glycaemic management in individuals with type 2 diabetes through effects on insulin secretion and weight loss [18]. The haemodynamic effect of endogenous GIP has yet to be investigated in individuals with type 2 diabetes. Additionally, it has been found recently that GIP contributes to ∼20% of the increased postprandial splanchnic blood flow in healthy individuals [3]. This raises the question of whether GIP also has an effect on splanchnic blood flow in individuals with type 2 diabetes.

Besides reduced insulin sensitivity and hyperglycaemia, the vascular system in general is severely affected in type 2 diabetes, with impaired angiogenesis, accelerated atherosclerosis and activation of inflammatory pathways, which promote complications involving both the micro- and macrovascular systems [19] and may lead to mesenteric ischaemia [20]. Additionally, diabetic autonomic neuropathy can reduce the intestinal postprandial blood flow response in line with chronic intestinal ischaemia [21]. Moreover, individuals with type 2 diabetes have a substantially increased burden of cardiovascular comorbidity, and cardiovascular disease remains a leading cause of mortality in this population.

In this single-blinded, crossover, placebo-controlled study, we investigated the importance of endogenous GIP for the postprandial splanchnic blood flow in individuals with type 2 diabetes using the selective GIP receptor antagonist GIP(3-30)NH_2_ [22]. Splanchnic blood flow was measured with MRI on four separate sessions, each including either oral water or oral glucose, and i.v. infusion of saline (154 mmol/l NaCl) or GIP receptor antagonist.

The hypothesis of this study is that the GIP-mediated physiological increase in splanchnic blood flow is significantly reduced or even absent in people with type 2 diabetes.

## Methods

### Approvals and registration

The study was performed at the Department of Clinical Physiology and Nuclear Medicine, Rigshospitalet, Copenhagen, Denmark. The Scientific Ethical Committee of the Capital Region of Denmark approved the protocol (ID no. H-20078806) and the Danish Data Protection Agency approved the handling of data (Capital Region of Denmark, P-2021-772). The study was registered at ClinicalTrials.gov (NCT06426823). The investigation was carried out in accordance with the principles of the declaration of Helsinki. All participants gave informed written consent to participate in the study.

### Participants

Ten participants with type 2 diabetes fulfilling the inclusion criteria of type 2 diabetes, age 20–80 years old, BMI 20–35 kg/m^2^, were included (Table 1). Exclusion criteria were treatment with medicine that could not be paused for 12 h, metallic implants incompatible with MRI, abuse of substances or alcohol, HbA_1c_ <48 mmol/mol (6.5%) or >75 mmol/mol (9.0%), alanine aminotransferase (ALAT) above two times normal value, decreased kidney function, unregulated hypertension, haemoglobin in the anaemic range, planned weight loss during the study period, blood vessels not suitable for measurements of flow, or any other condition that was not suitable for this study. The study sample included primarily Caucasian participants. Participants were aged 59–74 years from the capital region of Denmark. The sample may not fully represent the broader population because of their state of diabetes. In line with SAGER guidelines, sex was considered in the study design, which is why both men and female participants were included, and we did not expect difference in the role of GIP regarding splanchnic blood flow. Participants’ sex was determined by self-report.

**Table 1:** Participant characteristics

Characteristic	Mean	Range
No. of participants	10	
Age, years	66	59–74
Sex, *n* female / *n* male	2/8	
Height, cm	178	162–196
Weight, kg	88	77–107
BMI, kg/m^2^	28	23–35
Systolic BP, mmHg^a^	134	108–170
Diastolic BP, mmHg^a^	83	69–117
HbA_1c_, mmol/mol	55	49–62
HbA_1c_, %	7.2	6.6–7.8
Fasting blood glucose, mmol/l	6.3	4.3–10
Medication, *n*		
Metformin	6	
GLP-1 analogue	1	
SGLT2 inhibitor	3	
Metformin + SGLT2 inhibitor	3	
Antihypertensive medication	5	
Cholesterol-lowering medication	5	
Anticoagulant	5	
Antihistamine	1	
Proton pump inhibitor	1	
β_2_ bronchodilator	1	

^a^Data reflect *n*=9; the participant on GLP-1 analogue was on a stable initial dose while participating in the study

SGLT2, sodium–glucose cotransporter 2

### Study design

Participants were instructed not to consume alcohol or perform strenuous physical activity for 48 h before each session. The study participants received i.v. GIP(3-30)NH_2_ or saline infusions, and oral glucose or water in a randomised, single-blinded, crossover design. The order of the sessions was randomised following a randomisation code from http://www.random.org. The composition of the infusions was blinded for the study participants. All experiments were conducted in the afternoon and after 7 h of fasting. A cannula was placed in a cubital vein in both arms, one for infusion and one for blood samples. At time point −20 min, the infusion was started and at time point 0 min the oral glucose or water was ingested.

On the morning of each session, the participants were given a breakfast meal of a standardised 200 ml nutrient drink (1883 kJ [450 kcal] energy; 40% carbohydrate, 25% protein, 35% fat), if necessary supplemented by any desired oral liquid intake. During the 7 h fast, they were allowed to consume up to 200 ml of water.

### Data collection

**Fig. 1:**
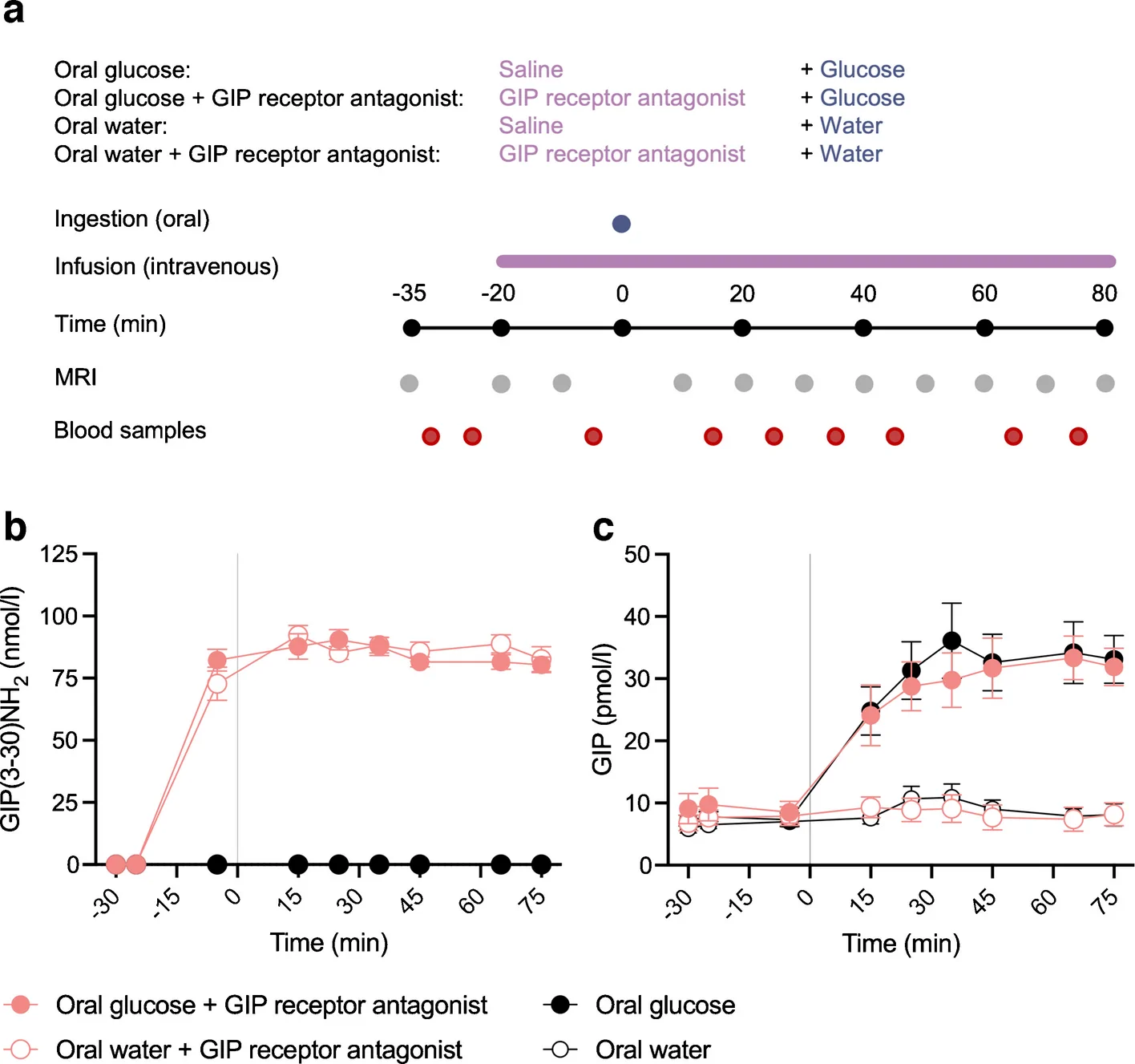
Study design and levels of GIP during study sessions. (**a**) Study design. (**b**) Plasma levels of GIP(3-30)NH_2_. (**c**) Plasma levels of GIP. Filled black circles, oral glucose; filled pink circles, oral glucose with GIP receptor antagonist; unfilled black circles, oral water; unfilled pink circles, oral water with GIP receptor antagonist. Values represent mean ± SEM. *n*=10. The vertical grey line indicates the time of ingestion (t=0 min)

Using MRI, blood flow measurements were collected at time points −35, −20 and −10 min before and at time 10, 20, 30, 40, 50, 60, 70 and 80 min after ingestion of either oral water or oral glucose. The infusion of saline or GIP receptor antagonist GIP(3-30)NH_2_ was started 20 min before ingestion of oral water or oral glucose to ensure equilibrium. Blood samples were collected at −30, −25 and −5 min before and at 15, 25, 35, 45, 65 and 75 min after ingestion of either oral water or oral glucose (Fig. 1a). Phase-contrast MRI was conducted as described in [3], using a 3 Tesla Siemens Biograph MRI (Siemens Healthineers, Erlangen, Germany). Images for T2* were acquired with a two-dimensional transaxial sequence covering the whole liver over three breath holds (field of view 285 × 380 mm^2^; voxel size 1.5 × 1.5 × 5 mm^3^; repetition time 293 ms; flip angle 60°; generalised autocalibrating partially parallel acquisitions acceleration factor 2). Seven echo times were acquired starting at 2.46 ms and with a spacing of 2.46 ms. Segmentation of the entire liver and gallbladder was performed using Total Segmentator on the T2* images and used for volume calculations (https://github.com/wasserth/TotalSegmentator). T2* was measured to investigate liver metabolism. Liver volume was investigated as a blood pooling control in relation to splanchnic blood flow measurements.

Blood was collected into chilled tubes with EDTA with a specific dipeptidyl-peptidase-4 (DPP-4) inhibitor (valine pyrrolidide [0.01 mmol/l], a gift from Novo Nordisk, Måløv, Denmark). All tubes were cooled on ice after sampling and were centrifuged for 7 min at ~2,000 *g* and stored at −20°C until analysis.

### Infusion

Synthetic peptide GIP(3-30)NH_2_ (CASLO, Kongens Lyngby, Denmark) were at least 97% pure and corresponded to the expected structure by mass spectrometry. GIP(3-30)NH_2_ was dissolved in a buffer of NaHCO_3_ (50 mmol/l), NaCl (9 mg/ml) and 0.5% human serum albumin (CSL Behring, Marburg, Germany). The dissolved peptide was sterile filtered. Vials with dissolved peptide were stored at −20°C and samples underwent microbiological evaluation after 3 weeks. Before each session, vials were thawed and prepared for infusion under sterile conditions after dilution to a total volume of 120 ml in sodium chloride (9 mg/ml; Braun, Melsungen, Germany). Infusion rates were 1000 pmol kg^−1^ min^−1^ for GIP(3-30)NH_2_ and 40 ml/h for saline.

### Blood samples

Whole-blood glucose concentration was measured at the bedside with an Accuchek Inform II (Roche Diagnostics, Mannheim Germany). GIP(3-30)NH_2_ was measured in plasma as previously reported [8, 23]. Plasma glucagon was measured by ELISA (10-1271-01; Mercodia Uppsala, Sweden). Total GIP concentration was measured with an in-house RIA using an antibody directed towards the C-terminal (code no. 80867) [23]. Insulin [24] and C-peptide were analysed with two-sided electrochemiluminescence assay (cobas, 800, e801; Roche, Mannheim Germany).

### Statistical analyses and calculations

Data are reported as mean ± SEM in figures and as means (95% CI) in tables as stated. For baseline correction, all data points were divided by the mean of the two baseline values (two first MRI results) and multiplied by 100. The calculations and statistical analysis were conducted in RStudio version 2023.12.0 + 369; https://posit.co with the packages lmertest, dplyr and ggplot2, and GraphPad Prism version 10.6.0 (796); https://www.graphpad.com. To evaluate the effects of the interventions and avoid sensitivity to false-positive error from repeated measurements, we used a linear mixed model. To address the primary endpoint, the effect of GIP(3-30)NH_2_ on the blood flow response to oral glucose, a single comparison was made between oral glucose ingestion during GIP(3-30)NH_2_ infusion vs oral glucose with a saline infusion. All timepoints were divided into two groups by each data point labelled as either pre-intervention or post-intervention (depending on the ingestion of either water or glucose). Three pre-intervention measures followed by eight post-intervention measures were acquired (Fig. 1a). To evaluate the effect of oral glucose or water on blood flow, models were constructed with a regressor indicating pre- or post-intervention as fixed effect and participant identification as a random effect to account for repeated measurements. The additional effect of GIP(3-30)NH_2_ infusion on blood flow response to oral glucose was tested in a model also including an interaction term between pre- and post-intervention regressor and a regressor indicating the session (GIP receptor antagonist vs saline).

## Results

Of 14 participants invited for screening, ten were included and completed the study. One was excluded due to being unable to go 12 h without smoking, one was excluded due to anatomical variation and two were excluded due to claustrophobia in the MRI scanner (electronic supplementary material [ESM] Fig. 1). For all included participants, the three vessels investigated were visualised during the screening. No adverse events occurred during the experiments. The baseline levels of GIP(3-30)NH_2_ were undetectable at baselines and during saline infusions (Fig. 1a). During infusion of GIP(3-30)NH_2_, plasma levels reached steady state after 15 min at ~85 nmol/l. Plasma levels of GIP rose similarly after oral glucose ingestion with and without GIP receptor antagonist infusion to ~30 pmol/l (Fig. 1c) and remained at stable fasting levels during ingestion of water (Fig. 1b, c).

### Splanchnic blood flow

#### Arteria mesenterica superior

**Fig. 2:**
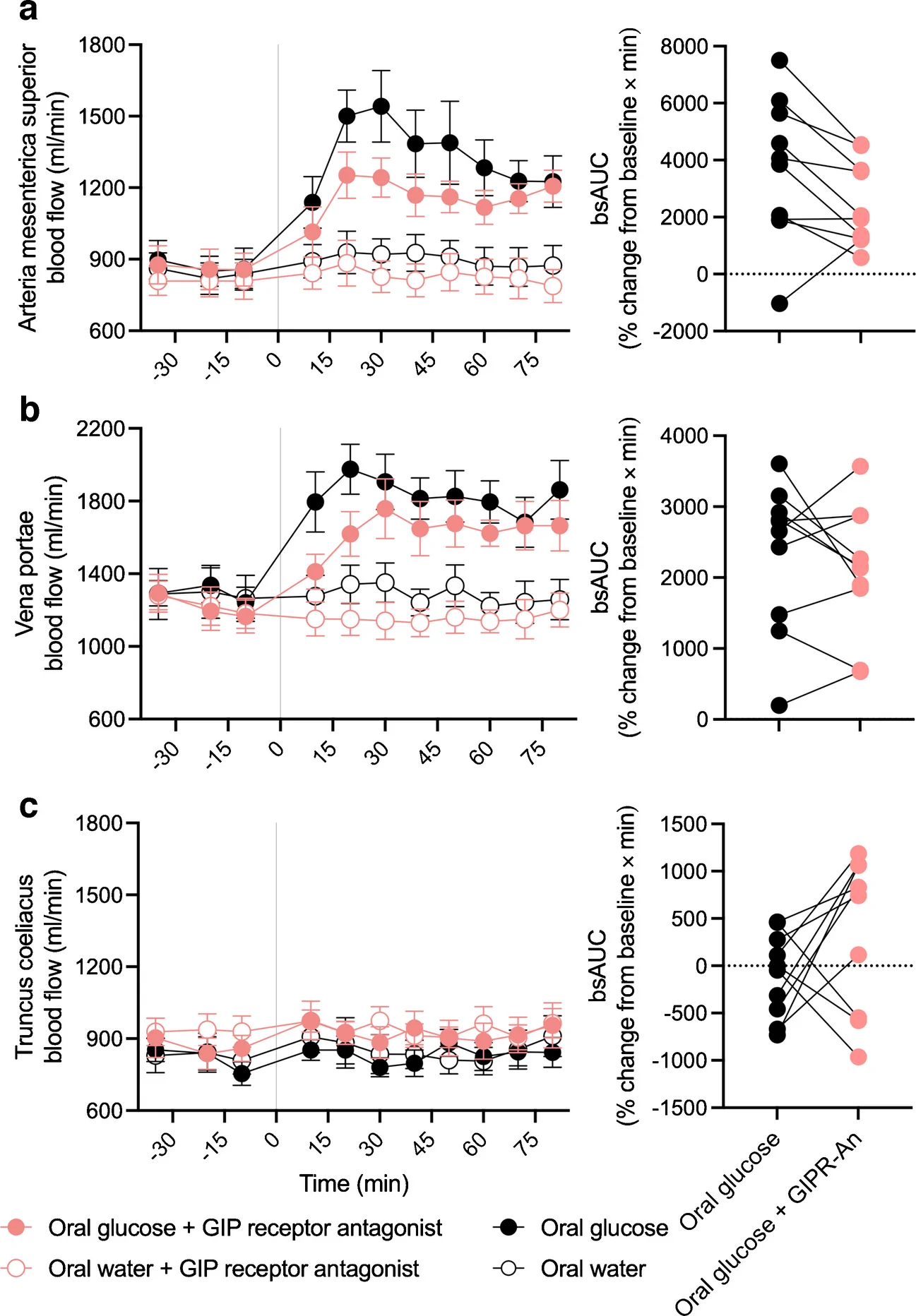
Splanchnic haemodynamic responses to oral glucose or oral water with and without GIP receptor antagonist infusion. (**a**) Blood flow in the arteria mesenterica superior. (**b**) Blood flow in the vena portae. (**c**) Blood flow in the truncus coeliacus. Filled black circles, oral glucose; filled pink circles, oral glucose with GIP receptor antagonist; unfilled black circles, oral water; unfilled pink circles, oral water with GIP receptor antagonist. *n*=10. Values represent mean ± SEM. The vertical grey line indicates the time of ingestion (t=0 min). bsAUC, baseline-subtracted AUC, from the time points 20 min to 80 min; GIPR-An, GIP receptor antagonist

Baseline measurements of blood flow were stable and similar at all sessions, at ~850 ml/min (Table 2). During oral water intake alone, or oral water combined with infusion of GIP receptor antagonist, blood flow was stable in the arteria mesenterica superior (Table 2, Fig. 2a). Following oral glucose ingestion, superior mesenteric artery blood flow increased by 57% (95% CI 26, 88) relative to baseline during the 20–80 min postprandial period (Fig. 2a). This rise in postprandial mesenteric blood flow was attenuated to 36% (95% CI 17, 55) of baseline flow during concomitant infusion of a GIP receptor antagonist over the same time interval. GIP(3-30)NH_2_ reduced blood flow by 15% (95% CI −2.0, 32; *p*=0.012 for oral glucose vs oral glucose + GIP receptor antagonist) for the whole period investigated, comparing GIP receptor antagonist infusion with placebo infusion during oral glucose. Increases in mesenteric artery blood flow were observed after oral glucose ingestion in all participants, except for one who did not display changes in either blood flow or blood glucose (ESM Figures 2, 3).

**Table 2:** Blood flow during oral water, oral water + GIP receptor antagonist, oral glucose, and oral glucose + GIP receptor antagonist (*N*=10 participants per group)

Blood vessel	Oral water	Oral water + GIP receptor antagonist	Oral glucose	Oral glucose + GIP receptor antagonist	*p* value
Arteria mesenterica superior					
Baseline, ml/min	840 [750, 930]	809 [724, 893]	874 [775, 973]	867 [755;979]	
Mean, ml/min	900 [844, 955]	829 [774, 884]	1365 [1269, 1461]	1186 [1130, 1242]	0.012
bsAUC, % of baseline × min	445 [−19, 909]	144 [−405, 694]	3658 [2107, 5208]	2470 [155, ;3382]	
Vena portae					
Baseline, ml/min	1295 [1102, 1488]	1251 [1126, 1377]	1315 [1201, 1429]	1243 [1100, 1387]	
Mean, ml/min	1285 [1211, 1358]	1153 [1089, 1217]	1838 [1738, 1937]	1665 [1570, 1760]	0.098
bsAUC, % of baseline × min	185 [−571, 941]	−380 [−1161, 401]	2329 [1688, 2971]	2100 [1534, 2666]	
Truncus coeliacus					
Baseline, ml/min	836 [732, 939]	933 [850, 1016]	846 [765, 927]	870 [769, 972]	
Mean, ml/min	775 [700, 851]	936 [896, 975]	833 [792, 873]	917 [865, 969]	0.313
bsAUC, % of baseline × min	42 [−742, 826]	63 [331, 459]	−90 [−361, 181]	375 [−122, 871]	

Data are mean [95% CI]

The *p* value was obtained from a linear mixed model comparing the interventions oral glucose vs oral glucose + GIP receptor antagonist

bsAUC, baseline-subtracted AUC, from time points 20 min to 80 min

#### Vena portae

The baseline blood flow in the vena portae was stable and similar at all sessions, at ~1280 ml/min (Table 2). During oral water ingestion, blood flow in the vena portae was stable both with and without GIP receptor antagonist infusion. Blood flow during oral glucose ingestion was approximately 46% (95% CI 24, 67) higher than with oral water alone (Table 2, Fig. 2b). The mean blood flow in the vena portae during oral glucose ingestion and concomitant GIP receptor antagonist infusion was 11% (95% CI 0.3, 22) lower compared with flow during oral glucose alone (*p*=0.098).

#### Truncus coeliacus

The baseline blood flow in the truncus coeliacus was stable and similar at all sessions, at ~870 ml/min (Table 2). There were no changes in blood flow during oral glucose ingestion with or without GIP receptor antagonist infusion (*p*=0.313; Table 2, Fig. 2c). Generally, the blood flow was stable in the truncus coeliacus during all interventions (Table 2, Fig. 2c).

### Heart rate

**Fig. 3:**
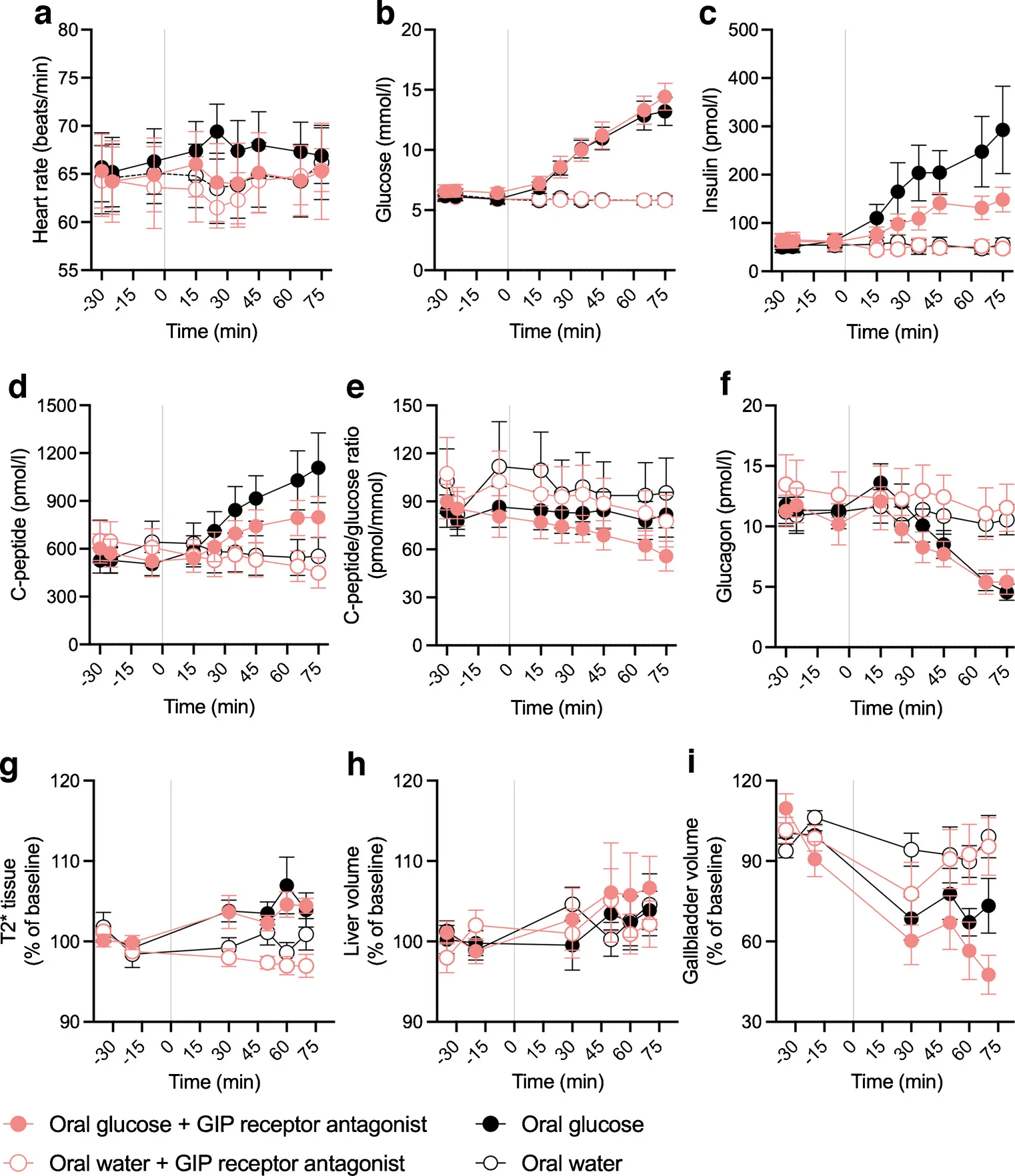
Glucometabolic markers, T2* tissue, hepatic volume, gallbladder volume and heart rate. (**a**) Heart rate. (**b**) Glucose. (**c**) Insulin. (**d**) C-peptide. (**e**) C-peptide/glucose ratio. (**f**) Glucagon. (**g**) T2* tissue. (**h**) Liver volume. (**i**) Gallbladder volume. Filled black circles, oral glucose; filled pink circles, oral glucose with GIP receptor antagonist; unfilled black circles, oral water; unfilled pink circles, oral water with GIP receptor antagonist. *n*=10. Values represent mean ± SEM. The vertical grey line indicates the time of ingestion (t=0 min)

The heart rates of the participants were similar before all interventions (Table 3, Fig. 3a). For the period 20–50 min the heart rates were significantly lower during GIP receptor antagonist infusion and oral glucose, compared with oral glucose and placebo infusion (*p*=0.001). For the whole period after the ingestion of glucose (20–80 min), the mean heart rate was not significantly lower during GIP receptor antagonist infusion than during placebo infusion (Fig. 3a, Table 3) (*p*=0.10).

**Table 3:** Glucometabolic markers, GIP levels and T2* measurements (*N*=10 participants per group)

Variable	Oral water	Oral water + GIP receptor antagonist	Oral glucose	Oral glucose + GIP receptor antagonist	*p* value
Insulin					
Baseline, pmol/l	140.5 [137.6, 143.4]	147.5 [136.9, 158.1]	75.0 [24.0, 125.9]	145.0 [123.4, 166.6]	
Mean, pmol/l	116.1 [74.7, 157.5]	68.4 [36.8, 99.9]	281.7 [212.3, 351.1]	168.6 [145.95, 191.3]	0.00088
AUC, pmol/l × min	2460 [1173, 3747]	2218 [1302, 3134]	10,441 [84,837, 16,045]	5955 [4086, 7825]	
C-peptide					
Baseline, pmol/l	591.9 [436.2, 747.6]	649.1 [482.99, 815.2]	527.9 [420.9, 634.9]	568.6 [448.1, 689.2]	
Mean, pmol/l	563.5 [460.5, 666.4]	512.2 [425.0, 599.4]	921.5 [775.7, 1067.2]	700.1 [610.0, 790.2]	0.0022
AUC, pmol/l × min	28,011 [16,235, 39,786]	25,834 [16,009, 35,658]	46,691 [31,021, 62,361]	14,461 [28,069, 45,995]	
C-peptide/glucose ratio					
Baseline, pmol/mmol	93 [70, 115]	99 [74, 123]	81 [68, 94]	88 [73, 102]	
Mean, pmol/mmol	95 [77, 113]	87 [73, 101]	82 [71, 93]	67 [59, 75]	0.0041
AUC, pmol/mmol × min	4750 [2700, 6800]	4370 [2840, 5890]	4090 [2930, 5250]	3350 [2580, 4110]	
Plasma glucose					
Baseline, mmol/l	6.2 [5.6, 6.9]	6.2 [5.7, 6.6]	6.2 [5.7, 6.6]	6.6 [5.9, 7.3]	
Mean, mmol/l	5.9 [5.6, 6.1]	5.8 [5.6, 6.1]	11.1 [10.2, 12.1]	11.5 [10.4, 12.6]	0.803
AUC, mmol/l × min	292 [258, 326]	292 [264, 319]	566 [473, 659]	583 [485, 681]	
Glucagon					
Baseline, pmol/l	11.1 [9.2, 12.9]	13.3 [10.1, 16.6]	11.5 [9.4, 13.7]	11.5 [9.7, 13.4]	
Mean, pmol/l	10.6 [9.5, 11.7]	12.0 [10.2, 13.8]	8.1 [6.9, 9.3]	7.3 [6.3, 8.4]	0.547
AUC, pmol/l × min	533 [416, 649]	601 [405, 797]	392 [297, 487]	355 [258, 452]	
GIP intact					
Baseline, pmol/l	6.2 [5.3, 7.1]	7.3 [5.6, 8.9]	7.5 [6.3, 8.7]	9.4 [6.0, 12.8]	
Mean, pmol/l	9.3 [7.8, 10.8]	8.3 [6.6, 9.9]	33.5 [29.4, 37.6]	31.1 [27.7, 34.5]	0.223
AUC, pmol/l × min	457 [312, 601]	403 [214, 592]	1685 [1225, 2145]	1576 [1229, 1924]	
GIP(3-30)NH_2_					
Baseline, nmol/l	0 [0, 0]	0 [0, 0]	0 [0, 0]	0 [0, 0]	
Mean, nmol/l	0 [0, 0]	85.1 [81.7, 88.5]	0 [0, 0]	84.5 [81.7, 87.3]	-
Heart rate					
Baseline, beats/min	65 [60, 69]	64 [59, 69]	66 [61, 70]	65 [59, 70]	
Mean, beats/min	65 [62, 68]	64 [61, 66]	68 [65, 70]	64 [61, 68]	0.10
AUC, beats/min × min	3226 [2878, 3573]	3194 [2924, 3464]	3385 [3081, 3689]	3083 [2680, 3486]	
T2* tissue					
Baseline, ms	12.5 [11.1, 13.8]	13.3 [12.3, 14.4]	13.0 [11.5, 14.4]	12.6 [11.5, 13.8]	
Mean, ms	12.7 [11.7, 13.7]	13.0 [12.2, 13.7]	13.3 [12.1, 14.5]	13.2 [12.3, 14.0]	0.526
bsAUC, % × min	−60 [−210, 91]	−186 [−316, −56]	336 [55, 616]	252 [37, 467]	
Liver volume					
Baseline, ml	1444 [1341, 1547]	1440 [1355, 1524]	1429 [1251, 1606]	1443 [1360, 1527]	
Mean, ml	1429 [1335, 1524]	1433 [1334, 1533]	1557 [1516, 1598]	1463 [1366, 1561]	0.515
bsAUC, % × min	199 [−36, 435]	157 [−232, 547]	51 [−190, 292]	280 [−274, 834]	
Gallbladder volume					
Baseline, ml	25.9 [22, 29.7]	25.5 [20.1, 30.7]	33.2 [28.9, 37.5]	26.1 [21.5, 30.7]	
Mean, ml	24.5 [20.9, 28]	21.2 [18.7, 23.7]	23 [20.2, 25.8]	15.2 [12.2, 18.2]	0.126
bsAUC, % × min	−261 [−975, 453]	−1052 [−2316, 212]	−1885 [−2780, −998]	−2847 [−4067, −1626]	

Data are mean (95% CI)

The *p* value was obtained from a linear mixed model comparing the interventions oral glucose vs oral glucose + GIP receptor antagonist

bsAUC, baseline-subtracted AUC, from time points 20 min to 80 min

### Glucometabolic markers

Glucose, insulin, C-peptide, and glucagon were similar at baseline during all sessions and reflected a fasting state (Table 3). Plasma glucose rose during oral glucose to 11.1 mmol/l (95% CI 10.2, 12.1), and to 11.5 mmol/l (95% CI 10.4, 12.6) during oral glucose and GIP receptor antagonist infusion with no difference between the two interventions (Fig. 3b, Table 3). Likewise, insulin and C-peptide rose during oral glucose intake (Fig. 3c, d, Table 3) and both C-peptide (*p*=0.002) and insulin (*p*<0.0001) were lower during GIP receptor antagonism compared with oral glucose alone (Fig. 3c, d, Table 3). The C-peptide/glucose ratio was stable during water ingestion, and higher during oral glucose intake. Likewise, GIP receptor antagonism attenuated the glucose-mediated increase in C-peptide/glucose ratio by 10% (95% CI −8, 28), reflecting the role of endogenous GIP in postprandial insulin secretion (*p*=0.004 for comparison between oral glucose and oral glucose + GIP receptor antagonist infusion) (Fig. 3e, Table 3). Glucagon levels decreased during oral glucose intake and were unaffected by GIP receptor antagonist infusion (Fig. 3f, Table 3).

### T2* measurements, hepatic volume and gallbladder volume

T2* values for liver tissue reflect hepatic oxygenation and/or perfusion. Baseline hepatic T2* values did not vary between sessions (Fig. 3g, Table 3). During oral water ingestion, the hepatic T2* values were stable. During oral glucose ingestion, T2* values were higher compared with oral water (*p*=0.018) (Fig. 3g). The GIP receptor antagonist infusion did not impact the T2* measurements. There were no changes in hepatic volume during the sessions or between interventions (Fig. 3h). Oral glucose compared with oral water resulted in a decrease in gallbladder volume (*p*=0.003). During the digestion period of oral glucose, gallbladder volume decreased by ~25% more during GIP receptor antagonist infusions when compared at time point 70 min. There was no difference during oral glucose with and without GIP receptor antagonist infusion measured from 20 min to 80 min (*p*=0.13, Fig. 3i).

## Discussion

On the basis of our previous investigations on splanchnic blood flow in healthy individuals and the poor effect of endogenous GIP in people with type 2 diabetes on metabolic markers [3, 14], we studied the role of endogenous GIP in individuals with type 2 diabetes. We found that endogenous GIP contributes, by approximately 15%, to the increased postprandial blood flow in the arteria mesenterica superior. These results are comparable with the role of endogenous GIP in healthy individuals for the same outcome (22% [95% CI 10, 35]) [3], and we conclude that the effect of endogenous GIP on splanchnic blood flow is conserved in individuals with type 2 diabetes despite the reduced glucose-lowering effect of GIP in these individuals [3, 14]. Impaired splanchnic blood flow regulation and postprandial hypotension is associated with the pathophysiology of diabetes where GIP could play a central role [25].

### Strengths and weaknesses

Due to the anatomical location and bowel movements, measurement of the splanchnic blood flow is challenging. Compared with doppler ultrasound, which measures mesenteric blood flow through the abdominal wall, problems with peristaltic movements can be avoided with MRI since the flow measures can be performed repeatedly near the aorta, which is stationary. Thus, it is possible to perform repeated blood flow measurements after the ingestion of, for example, glucose and have individuals participate in several successive visits, since radioisotopes or contrast media are not required for phase-contrast MRI. Unlike sitting or standing after a meal, the participant must be stationary in the supine position during the scan, which may influence the haemodynamic changes. Since the MRI scanner was also in clinical use all experiments were performed in the afternoon, with the consequence of the circadian rhythm affecting metabolic outcomes. Moreover, both temperature and nutrient composition will impact splanchnic blood flow so the oral administration of a chilled oral solution may not be representative of the common postprandial state [1]. We examined both sexes in this study, and we did not expect GIP to have different effects across the sexes consistent with the observations.

One participant had a very slow gastric emptying rate compared with the rest of the participants, resulting in a low and slow increase in blood glucose and splanchnic blood flow 60 min after oral glucose ingestion. The results excluding this participant are presented in ESM Figures 2, 3. The participant remained in the statistical analysis.

### Postprandial changes in splanchnic blood flow

Absolute values for the splanchnic blood flow presented in this study are similar to previous measurements, although previous measurements using a 1.5 T MRI scanner showed the absolute arteria mesenterica superior blood flow for healthy young and elderly individuals to be lower at rest than the values presented in Table 2 [26, 27]. In the present dataset, the resting blood flow in arteria mesenterica superior averaged approximately 850 ml/min (Table 2), whereas the results from previous measurements span from 374 ml/min to 739 ml/min. However, we found that young healthy individuals (age 20–46 years) had a mean resting blood flow in arteria mesenterica superior of 681 ml/min, ranging from 657 ml/min to 739 ml/min [3], compared with older individuals with type 2 diabetes (age 59–74 years) in whom the mean blood flow was 848 ml/min, ranging from 809 ml/min to 874 ml/min. We interpret these observations to mean that baseline splanchnic blood flow may vary with pathological glucometabolic regulation, which is a novel observation for individuals with type 2 diabetes.

Confirming previous findings [21, 28, 29], the higher postprandial blood flow seems to be vessel specific, not affecting truncus coeliacus, and mainly occurring in the arteria mesenterica superior and the portal vein. The latter reflects, at least partly, the increase in arteria mesenterica superior blood flow. This points to a vessel-specific regulation of blood flow in the postprandial state, which in general aligns with other observations where ultrasound was used to assess the blood flow in truncus coeliacus [28]. Someya et al report that blood flow in the truncus coeliacus only increases briefly during meal intake and returns to baseline 5 min after the ingestion of a meal (at which time point, we did not assess blood flow) [28].

An increase in the blood flow on a macrovascular level is only possible if the microvascular vessels allow the blood to be distributed in the tissue, passing the resistance vessels, in agreement with the relationship between flow and resistance at constant pressure (Q=Δ P/R, where Q is flow, P is pressure and R is resistance) [30]. The arterioles in close proximity to the capillary beds of the tissues are therefore the key to regulation of the blood flow and perfusion [30, 31]. From previous investigations, we know that GIP has the ability to recruit capillaries in humans [11].

We report blood flow changes in the postprandial period with serial measurements in individuals with type 2 diabetes. Our data suggest that despite the presence of insulin resistance, the haemodynamic effect of GIP receptor activation in the splanchnic circulation, also reflected by the effect on heart rate, remains functionally intact, supporting a role for GIP in postprandial haemodynamic regulation in people with type 2 diabetes. Future studies will reveal whether pharmacological modulation of the GIP receptor (e.g. through dual GLP-1 receptor-GIP receptor agonist treatment, such as tirzepatide) influences splanchnic blood flow dynamics and thereby contributes to improved postprandial haemodynamic regulation in individuals with type 2 diabetes.

### Effect of endogenous GIP on gallbladder volume, hepatic volume and hepatic oxygenation

Endogenous GIP has been implicated in regulating splanchnic functions, including gallbladder motility. In healthy individuals, GIP appears to influence gallbladder relaxation, making these endpoints physiologically relevant when assessing splanchnic responses in individuals with type 2 diabetes [32]. During oral glucose ingestion, GIP receptor antagonism appeared to effect gallbladder contraction (Fig. 3i) suggesting that endogenous GIP contributes to gallbladder relaxation. Previously, in healthy individuals, gallbladder volume was also slightly affected during GIP(3-30)NH_2_ infusion [32]. This effect might be more pronounced during mixed-meal ingestion, which causes a stronger gallbladder contraction [33].

We assessed hepatic metabolism using T2* hepatic measurements, which indirectly reflect the perfusion-dependent oxygenation in the liver tissue [34]. The T2* rose during oral glucose ingestion probably because of the increased blood flow and hence oxygen delivery (Fig. 3g) aligning with previous findings [3]. Liver volume was similar to that previously observed in healthy individuals and did not change with the interventions (Fig. 3h). The reason for including liver volume data was to control for blood pooling, which could affect the oxygenation measurements. With stable liver volume, the higher T2* during oral glucose ingestion compared with water indicates either a change in oxygenation, a change in perfusion, or a combination of the two. This explorative outcome was not affected by the GIP receptor antagonist infusions.

### GIP receptor targeting in obesity and type 2 diabetes

The dual receptor agonist tirzepatide is approved for treatment of type 2 diabetes and obesity [35]. Moreover, several drugs in late-stage clinical development target the GIP receptor as agonists or antagonists, often in combination with GLP-1 receptor agonism [18, 36, 37].

While GLP-1 receptor agonists promote satiety, delay gastric emptying and enhance insulin secretion in individuals with type 2 diabetes [38], the metabolic actions of GIP are less well defined, although GIP may exert both lipogenic and lipolytic actions [39]. Whether the additive benefit of GIP in dual receptor agonist therapies is partially mediated by its influence on splanchnic blood flow is yet to be elucidated. Increased splanchnic blood flow after food ingestion may facilitate nutrient absorption [40] and gut hormonal signalling, potentially amplifying the metabolic response to GIP. In our study, GIP receptor antagonism resulted in a ~15% smaller increase in superior mesenteric blood flow, underscoring the vascular contribution of GIP to postprandial physiology in individuals with type 2 diabetes [3]. From animal studies, it is established that enhanced splanchnic blood flow increases intestinal metabolism estimated from oxygen uptake [40, 41]. Both treatment strategies, GIP receptor agonism and antagonism, result in lower body weight and improve glycaemic management in preclinical and clinical settings, and the debate as to whether GIP receptor agonists or antagonists are superior continues [36, 42, 43]. The mode of action of GIP in the human brain remains unknown but studies in mice suggest that the regulation of body weight through GIP receptors is exerted via the central nervous system [44]. There are several suggestions to explain the blunted effect of exogenous GIP on glucose regulation in individuals with type 2 diabetes. One is that glucotoxicity impairs GIP receptor signalling [45], another is increased internalisation and desensitisation of the GIP receptors leading to the dysfunction of the pancreatic beta cells [46]. The GIP receptor pharmacology is complex, and differs between species, suggesting that results from animal studies cannot be translated into humans [47].

For the human GIP system, it is essential to distinguish between the physiological effects of endogenous GIP and the pharmacological actions of exogenous GIP in individuals with type 2 diabetes. While the incretin effect of exogenous GIP appears to be blunted in individuals with type 2 diabetes, endogenous GIP remains physiologically relevant. Thus, also using the GIP receptor antagonist GIP(3-30)NH_2_, glucose-adjusted insulin secretion assessed by C-peptide/glucose ratio was ~14 ± 6.5% lower than with placebo [14], supporting its relevance in individuals with type 2 diabetes [14, 48]. During oral glucose ingestion in our study, plasma glucose rose as expected but there was no difference between glucose levels measured during GIP receptor antagonist infusion or placebo (Fig. 3b), although insulin, C-peptide and C-peptide/plasma glucose ratio were significantly influenced by endogenous GIP. Similar results were reported previously [14], and support a minor postprandial, glucometabolic role for GIP in individuals with type 2 diabetes.

### Conclusion

Similarly to the case in healthy individuals, GIP contributes to increased splanchnic blood flow in individuals with type 2 diabetes.

## Supplementary Information

Below is the link to the electronic supplementary material.

ESM Figs (PDF 278 KB)

## Data Availability

The datasets generated during the current study are available from the corresponding author. Access will be granted to qualified researchers for non-commercial research purposes, subject to approval by the corresponding author and, where applicable, institutional or ethical guidelines. Requests should include a brief description of the proposed research and analysis plan. There are no time restrictions on availability, provided that data storage remains compliant with institutional policies. Data will not be shared if this would violate participant confidentiality or consent agreements. The software for calculating blood flow PCM techniques is available at https://github.com/MarkVestergaard/PCMCalculator. Liver and gallbladder volumes were determined using Total Segmentator (https://github.com/wasserth/TotalSegmentator). The MRI images are not publicly available due to privacy restrictions.

## Abbreviations

GIP: Glucose-dependent insulinotropic polypeptide
GLP-1: Glucagon-like peptide 1
