# Supplementary material for: GIP contributes to postprandial regulation of splanchnic blood supply in humans with type 2 diabetes: a randomised, single-blinded, placebo-controlled, crossover study
Source: Diabetologia. 2026 Jul 2;69(10):2897–910. doi: 10.1007/s00125-026-06788-1 (PMC13541893; doi:10.1007/s00125-026-06788-1)
Supplement: Supplementary file 1 [file 125_2026_6788_MOESM1_ESM.pdf]

Electronic supplementary material (ESM)

Supplementary Figure 1

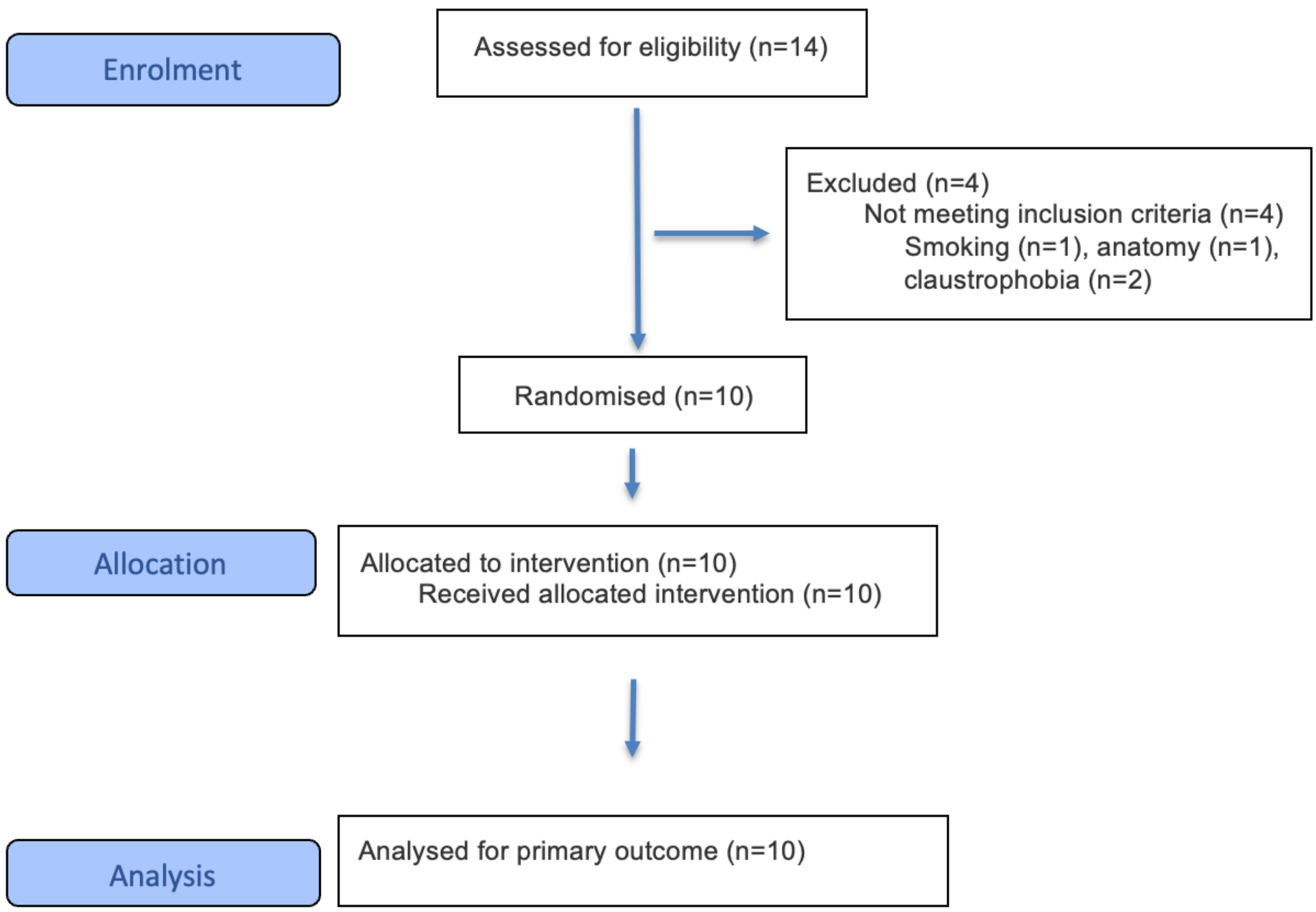

Supplementary Figure 1: Flow chart. Participant exclusion, randomisation, allocation and completed participants.

Supplementary Figure 2

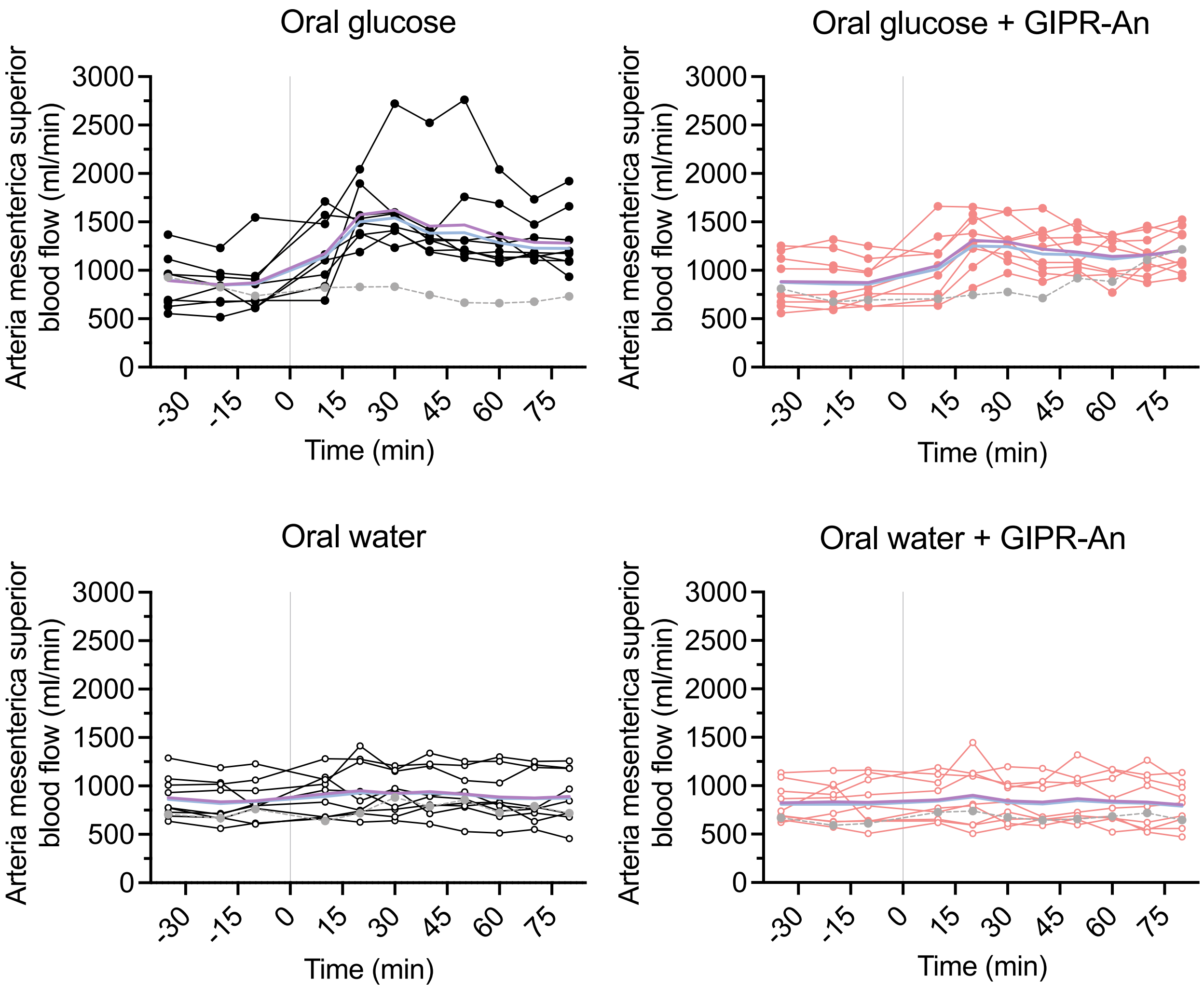

Supplementary Figure 2: Individual blood flow measurement during the four sessions. Oral glucose: Oral glucose ingestion at timepoint 0, saline infusion from timepoint -20 min to 80 min. Oral glucose + GIPR-An: Oral glucose ingestion at timepoint 0, GIP(3-30)NH_2 infusion from -20 min to 80 min. Oral water: Oral water ingestion at timepoint 0, saline infusion from -20 min to 80 min. Oral water + GIPR-An: Oral water ingestion at timepoint 0, saline infusion from -20 min to 80 min. Purple line shows mean blood flow without the participant with slow gastric emptying. Blue show mean blood flow of all participants. Curve with points show individual measurements for ten participants. Grey filled circles with striped line show the individual with slow gastric emptying. GIPR-An; Glucose dependent insulinotropic polypeptide receptor antagonist (GIP(3-30)NH_2).

Supplementary Figure 3

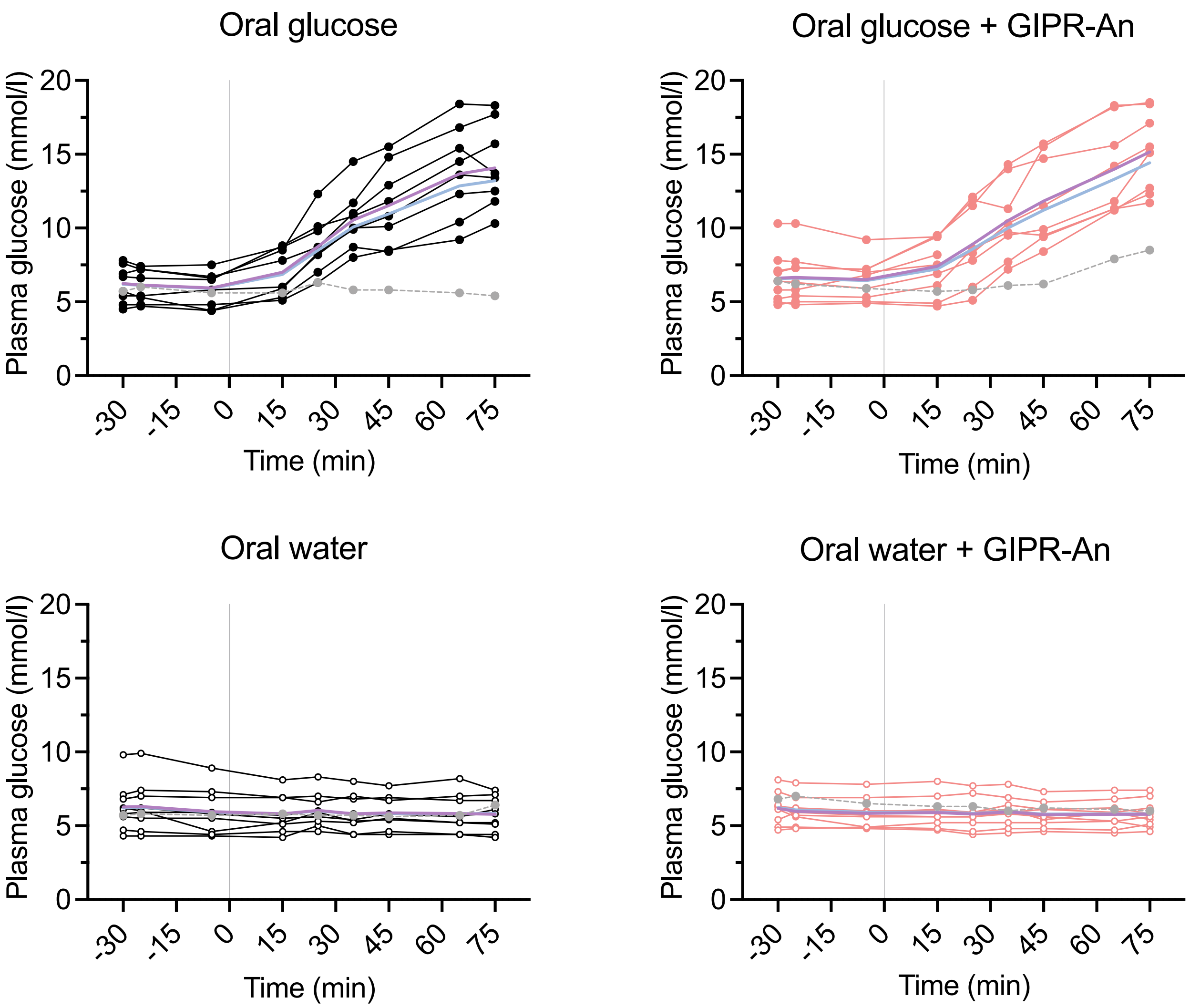

Supplementary Figure 3: Individual measurement of blood glucose during four sessions. Oral glucose: Oral glucose ingestion at timepoint 0, saline infusion from timepoint -20 min to 80 min. Oral glucose + GIPR-An: Oral glucose ingestion at timepoint 0, GIP(3-30)NH_2 infusion from -20 min to 80 min. Oral water: Oral water ingestion at timepoint 0, saline infusion from -20 min to 80 min. Oral water + GIPR-An: Oral water ingestion at timepoint 0, saline infusion from -20 min to 80 min. Purple line shows mean plasma glucose without the participant with slow gastric emptying. Blue line shows mean plasma glucose of all participants. Black / red circles show individual measurements for ten participants. Grey filled circles with striped line show the individual with slow gastric emptying. Five plasma samples are missing from one individual.
